# Caffeine Increases Anaerobic Work and Restores Cycling Performance following a Protocol Designed to Lower Endogenous Carbohydrate Availability

**DOI:** 10.1371/journal.pone.0072025

**Published:** 2013-08-19

**Authors:** Marcos David Silva-Cavalcante, Carlos Rafaell Correia-Oliveira, Ralmony Alcantara Santos, João Paulo Lopes-Silva, Hessel Marani Lima, Romulo Bertuzzi, Marcos Duarte, David John Bishop, Adriano Eduardo Lima-Silva

**Affiliations:** 1 Sports Science Research Group, Faculty of Nutrition, Federal University of Alagoas, Maceió, Alagoas, Brazil; 2 Endurance Performance Research Group, School of Physical Education and Sport, University of São Paulo, São Paulo, São Paulo, Brazil; 3 Nephrology Division, Department of Medicine, Federal University of São Paulo, São Paulo, São Paulo, Brazil; 4 Faculty of Biomedical Engineering, Federal University of ABC, Santo André, São Paulo, Brazil; 5 Institute of Sport, Exercise and Active Living (ISEAL), Victoria University, Melbourne, Victoria, Australia; Wageningen University, The Netherlands

## Abstract

The purpose this study was to examine the effects of caffeine ingestion on performance and energy expenditure (anaerobic and aerobic contribution) during a 4-km cycling time trial (TT) performed after a carbohydrate (CHO) availability-lowering exercise protocol. After preliminary and familiarization trials, seven amateur cyclists performed three 4-km cycling TT in a double-blind, randomized and crossover design. The trials were performed either after no previous exercise (CON), or after a CHO availability-lowering exercise protocol (DEP) performed in the previous evening, followed by either placebo (DEP-PLA) or 5 mg.kg^−1^ of caffeine intake (DEP-CAF) 1 hour before the trial. Performance was reduced (−2.1%) in DEP-PLA vs CON (421.0±12.3 vs 412.4±9.7 s). However, performance was restored in DEP-CAF (404.6±17.1 s) compared with DEP-PLA, while no differences were found between DEP-CAF and CON. The anaerobic contribution was increased in DEP-CAF compared with both DEP-PLA and CON (67.4±14.91, 47. 3±14.6 and 55.3±14.0 W, respectively), and this was more pronounced in the first 3 km of the trial. Similarly, total anaerobic work was higher in DEP-CAF than in the other conditions. The integrated electromyographic activity, plasma lactate concentration, oxygen uptake, aerobic contribution and total aerobic work were not different between the conditions. The reduction in performance associated with low CHO availability is reversed with caffeine ingestion due to a higher anaerobic contribution, suggesting that caffeine could access an anaerobic “reserve” that is not used under normal conditions.

## Introduction

The importance of endogenous carbohydrate (CHO) availability for high-intensity exercise performance has been well described in the literature [1]–[4]. Several studies [1]–[5] have shown that performance during high-intensity exercise is impaired when endogenous CHO availability (i.e., muscle and liver glycogen stores) is reduced. For example, Langfort et al. [2] reported that after three days of a low-CHO diet (∼ 5% CHO) the average power output measured during a 30-s Wingate test in healthy men not engaged in any competitive sport was significantly reduced (from 581±7 to 533±7 W) when compared with a normal diet (∼ 50% CHO). According to these authors, the reduction in performance (∼9%) with low CHO availability was due to a lower contribution of the anaerobic energy system. Similarly, Miura et al. [5] found a reduction in the anaerobic work capacity of healthy, non-athletic men when exercise was performed after a muscle-glycogen-depletion protocol compared to a control condition (10.33±2.41 vs 12.83±2.21 kJ, respectively), suggesting that low CHO availability can reduce the anaerobic contribution to total energy expenditure during high-intensity exercise. In addition, reduction in self-selected power output during high-intensity interval training when performed with low endogenous CHO availability has been also reported in well-trained subjects, and it may be associated with a reduction in the anaerobic contribution [6]–[8].

While low CHO availability seems to reduce the anaerobic contribution and impair performance during high-intensity exercise, acute ingestion of caffeine seems to have the opposite effect [9], [10]. Doherty [10] observed an increase of 10% and 14% in the anaerobic energy supply (measured by maximum accumulated oxygen deficit) and performance (time to exhaustion), respectively, during high-intensity exercise performed at 125% of the maximal oxygen uptake (VO_2_max) after caffeine ingestion (5 mg.kg^−1^). Using the same exercise intensity and caffeine dose, Bell et al. [11] reported a 7–8% increase in total anaerobic energy contribution and time to exhaustion. Recently, a similar increase in the anaerobic contribution (6.5%) and time to exhaustion (14%) at 120% VO_2_peak was also found by Simmonds et al. [12] after caffeine ingestion (5 mg.kg^−1^). Although all of the aforementioned studies have investigated the effects of caffeine on performance during time-to-exhaustion tests, some studies have also found a positive effect of caffeine on performance during time-trials [13]–[15]; however, anaerobic contribution has not been measured in these studies. Taken together, these findings suggest that acute ingestion of caffeine may improve performance during high-intensity exercise via an increase in the anaerobic contribution.

The mechanisms by which caffeine increases the anaerobic contribution and performance during high-intensity exercise is not fully understood, but it has been proposed that caffeine intake would promote an inhibitory action on adenosine receptors, which would increase the activity of the enzyme phosphofructokinase, thereby increasing anaerobic glycolysis [12], [16]. Alternatively, caffeine may act on the central nervous system leading to an increase in motivational drive and neuromuscular excitability, which, in turn, results in a lowered rating of perceived exertion (RPE) for a given workload [17] and improved neuromuscular function, as measured via electromyography activity (EMG) [18]. In addition, it has also been suggested that caffeine attenuates muscle sensory signals to the brain and decreases the threshold of activation of motor neurons [19]. All of these central alterations could lead to an ability to produce more work anaerobically.

Although several studies have investigated the isolated effects of both CHO availability and caffeine intake on anaerobic contribution and performance [1]–[3], [10]–[12], no study has examined whether acute caffeine ingestion could counteract the negative effects of low CHO availability on both the anaerobic contribution and performance. This seems to be particularly important since many athletes perform two training sessions in the same day, or participate in multi-stage event races (e.g., *tour de France*), where the time to replenish endogenous CHO stores between sessions or races may not be sufficient. Furthermore, most studies with either caffeine [10]–[12] or CHO availability [1]–[3] have focused on investigating their effects during time-to-exhaustion tests. However, time trials (TT) appear to be more reliable [20] and to have greater external validity [21] compared to constant-workload tests until exhaustion. Furthermore, during a high-intensity TT, where athletes are free to vary power output, anaerobic metabolism seems to exert a decisive effect on both performance [22] and the distribution of work [23]–[25].

Therefore, the purpose of this study was to examine whether caffeine ingestion promotes an increase in the anaerobic contribution and performance during a 4-km cycling TT, when athletes begin the trial after reduced endogenous CHO availability [26]–[28]. We hypothesized that low CHO availability would reduce the anaerobic contribution and performance during a 4-km cycling TT; however, this reduction in the anaerobic contribution and performance would be attenuated or even reversed with caffeine supplementation.

## Methods

### Participants

Before beginning the study, participants answered a questionnaire on readiness for physical activity (PAR-Q) and performed a cardiac screening test, including electrocardiogram. Seven amateur cyclists (age 32.3±5.4 years, body mass 73.6±7.4 kg, height 173.1±5.3 cm, body fat 10.5±4.7%, peak power output [PPO] 227.8±10.2 W, and VO_2_peak 58.1±6.3 mL.kg^−1^.min^−1^) participated in this study. The participants had been training for at least 4 years, performed a weekly training volume of 12.1±4.3 h.wk^−1^ (250±101.3 km.wk^−1^) and competed regularly (∼20 competitions per year). The sample size required was estimated from the equation n = 8e^2^/d^2^, as suggested by Hopkins [29], where *n*, *e*, and *d* denote predicted sample size, coefficient of variation, and the magnitude of the treatment effect, respectively. Coefficient of variation was assumed to be 0.9% [20]. Expecting a magnitude of effect for the treatment of 3.1% [13], detection of a very conservative 1% difference as statistically significant would require at least 6 participants. The participants were informed about the risks associated with the study protocol and signed a consent form agreeing to participate in the experiments. This investigation was approved by the Ethics and Research Committee of the Federal University of Alagoas.

### Experimental Design

Each athlete visited the laboratory on seven different occasions. On the first visit, athletes underwent an anthropometric assessment and an incremental test. During the second visit (after ∼48 h), athletes performed a 4-km cycling familiarization trial. During the next visits, the athletes performed three 4-km cycling TT in a randomized, double-blind and repeated-measures crossover design, separated by 7 days for washout, under the following conditions: 1) 12–14 h after a validated exercise-protocol designed to reduce endogenous CHO availability, followed by placebo (DEP-PLA) or caffeine (5 mg.kg^−1^, DEP-CAF) ingestion one hour before the trial and 2) 12–14 h after a full-rest, followed by placebo ingestion one hour before the trial (CON). In the evenings before DEP-PLA and DEP-CAF trials, two additional visits were required and the athletes came to the laboratory to perform a validated protocol for reducing the endogenous CHO availability. All of the experimental trials were performed in the morning to avoid the impact of circadian variation [30], [31]. The athletes were asked to refrain from vigorous physical activities, caffeine and alcohol 24 h before each test. The temperature and relative humidity during the trials were 23.2±1.5°C and 43.1±4.2%, respectively.

### Incremental Test

The incremental test was performed on a cycle ergotrainer (Tacx™ T1680 Flow, Netherlands) and consisted of a 3-min warm-up at a power output (PO) corresponding to 100 W, followed by increments of 30 W every 3 min until voluntary exhaustion or when the athletes were not able to maintain pedal frequency between 80–90 rpm [32]. The ventilation (VE), VO_2_ and carbon dioxide production (VCO_2_) were measured breath-by-breath throughout the test with a gas analyzer (Quark CPET, Cosmed, Rome, Italy). The gas analyzer was calibrated before each test in accordance with the manufacturer’s recommendations. The HRmax and VO_2_peak were taken as the highest value reached in the last stage and as the mean value obtained during the last 30 s of the test, respectively. The PPO was determinate as highest PO maintained during a complete stage. When the last stage was not completed, the PPO was determined in according the methods of Kuipers et al. [33].

### Familiarization Trial

Forty-eight hours after the incremental test, the athletes performed a familiarization session with the TT procedures (cycle ergotrainer, Tacx™ T1680 Flow, Netherlands). The seat was fully adjustable vertically and horizontally for each cyclist before the TT, and cycling shoes were used to secure the feet to the pedals. The seat position was recorded and replicated during all subsequent experimental sessions. The athletes were instructed to perform the TT in the shortest possible time and to remain seated throughout test. The gear ratio was standardized at the beginning of each TT (53×16). However, immediately after the TT had started, participants were free to change the gear and pedal frequency as desired. The athletes were instructed to record all foods (type, amount and hour) consumed in the 48 hours before the familiarization test [34].

### Time-trials

**Figure 1:**
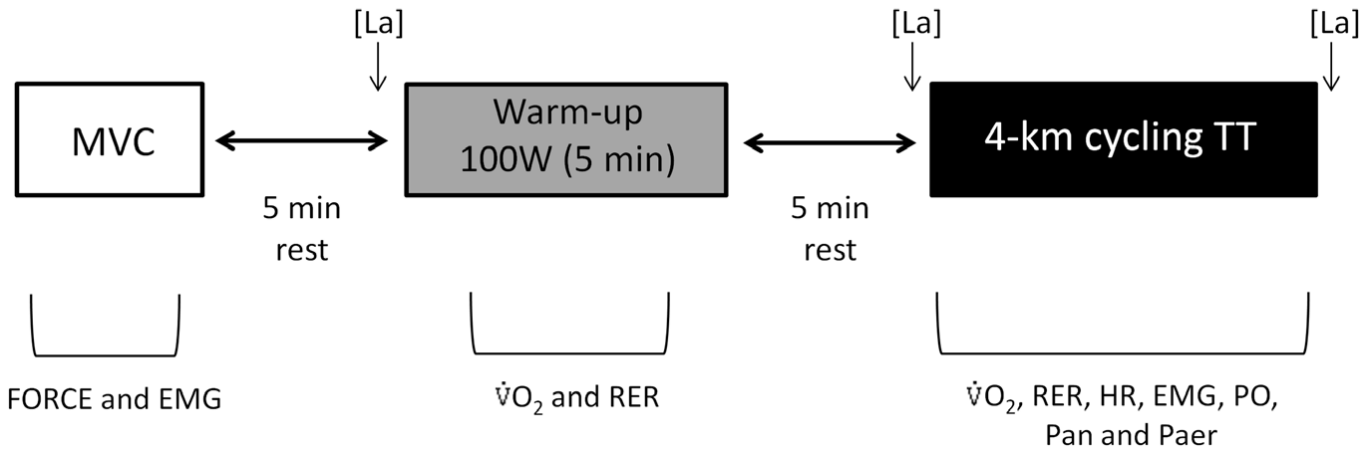
Experimental Protocol. [La]: plasma lactate concentrations; MVC: maximal voluntary contraction; EMG: electromyographic activity; VO_2_: oxygen uptake; RER: respiratory exchange ratio; HR: heart rate; PPO: power output; Paer: aerobic mechanical power output; Pan: anaerobic mechanical power output.

The TT sequence began ∼96 hours after the familiarization test. Before each trial, the cycle ergotrainer was calibrated in accordance with the manufacturer’s recommendations. The TT was preceded by three maximal voluntary contractions (MVC) of the quadriceps muscles and a 5-min warm-up at 100 W (90 rpm) followed by a 5-min rest period (Figure 1). The distance and PO were recorded at a frequency of 1 Hz (Tacx Trainer software 3.0, Wassenaar, Netherlands). Feedback of the distance covered was provided verbally every 200 m of the TT. The RPE was recorded every 1 km using the Borg 15-point category scale [35]. The VO_2_, respiratory exchange ratio (RER), HR, electromyographic activity (EMG), aerobic and anaerobic mechanical power output (Paer and Pan, respectively), work total (Wtot), total aerobic (Waer) and anaerobic work (Wan) were determined every 200 m and pooled for each 1 km. Blood samples (25 µL from an ear lobe) were obtained before the warm-up (rest), immediately before (Pre-TT) and 1 min after the TT (post-TT) to determine the plasma lactate concentrations [La]. The blood samples were transferred into micro-tubes containing sodium fluoride (NaF 1%) and then centrifuged at 3000 rpm at 5°C for 10 minutes. Plasma [La] was measured through enzymatic colorimetric reactions in a spectrophotometer (model Q798U2V5, Quimis®, São Paulo, Brazil) using commercial kits (kit Biotecnica, Varginha, Brazil).

### Calculations of Aerobic and Anaerobic Mechanical Power Output

The Pan and Paer were calculated from RER, VO_2_ and using gross mechanical efficiency estimated during the warm up, in accordance with Hettinga et al. [25]. Briefly, metabolic aerobic power (Pmet) during the warm up and TT was calculated by multiplying VO_2_ with the oxygen equivalent using the following equation:
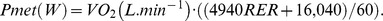



We assumed a RER equal to 1.00 during the time trials [25]. Gross mechanical efficiency was determined during the warm up, by dividing external PO (i.e. 100 W) by calculated Pmet. Aerobic mechanical power output (Paer) during the time trial was calculated by multiplying Pmet by gross efficiency. Anaerobic mechanical power output was calculated by subtracting the calculated Paer from the total measured mechanical PO.

### CHO-availability-lowering Exercise Protocol

Participants arrived at the laboratory at ∼8∶00 PM, at least two hours after their last evening meal. The protocol employed for reducing the endogenous CHO availability has previously been validated and has been shown to reduce endogenous CHO availability to ∼30% of pre-exercise values [26]–[28]. The protocol consisted of a constant PO exercise at an intensity corresponding to 70% PPO (159.9±7.0 W) for 90 min. After 5 min of rest, the athletes performed six 1-min exercise bouts at 125% PPO (285.1±13.0 W) interspersed with 1-min rest periods. During the protocol, the pedal frequency was maintained between 80–90 rpm.

### Dietary Control

During the morning and afternoon of the CHO availability-lowering exercise protocol, participants followed, up to the beginning of the exercise, the same dietary pattern contained in their food record. However, after the exercise protocol was finished (∼ 10∶00 PM), the athletes received a low-CHO diet (total energy 710.4±28 kcal, 12.7±0.1% CHO, 60.6±0.1% fat and 26.7±0.1% protein). Furthermore, the athletes received the same standardized, low-CHO breakfast (total energy 710.4±28 kcal, 12.7±0.1% CHO, 60.6±0.1% fat and 26.7±0.1% protein) one hour before the trial in the next morning (∼ 8∶00 AM). In the CON trial, the athletes were asked to replicate the diet recorded 24 hours before the trial, and ate a standardized meal derived from their diet record (total energy 737.0±103.7 kcal, 55.8±21.4% CHO, 26.5±14.7% fat and 17.7±7.8% protein). The breakfast consumed an hour before the CON trial was also derived from the diet recorded and consisted of 55.8±21.4% CHO, 26.5±14.7% fat and 17.7±7.8% protein (total energy 737.0±103.7 kcal).

### Maximal Isometric Contraction Measurement

Before the MVC, participants performed a standardized warm-up, in a chair with the trunk-thigh angle at 90° and the knee at 60° from full leg extension (0°), consisting of four 5-s contractions of the knee extensors, interspersed by 30-s rest periods, at intensities corresponding to 50, 60, 70 and 80% of the maximum subjective force [36]. Thereafter, participants performed three 5-s MVC (two legs), each separated by 60-s intervals. The peak force produced by the quadriceps muscles was recorded using a load cell (EMG System of Brazil, São José dos Campos, Brazil). The athletes were verbally encouraged during all MVCs to achieve their maximal force.

### Acquisition and Analysis of the Electromyographic Signal

During the MVC and time trials, EMG signals of the vastus lateralis (VL) muscle of the right leg were recorded via bipolar Ag-AgCl surface electrode (Hal, São Paulo, Brazil) at an interelectrode distance of 20 mm. We chose the VL muscle because it has been reported as the most appropriate to monitor EMG activity in the lower limb during a 4-km cycling TT [24]. The reference electrode was placed over the anterior surface of the tibia. The skin preparation, placement and location of the electrodes were in accordance with the recommendations of SENIAM [37]. To prevent movement artifact, the electrode wires were taped to the skin using adhesive tape (MicroporeTM 3 M, São Paulo, Brazil). Five seconds of raw EMG signal was recorded each 200 m with a sample rate of 2000 Hz (model 410c EMG System of Brazil Ltda, São Paulo, Brazil). Raw EMG signals were full-wave rectified and filtered with second-order Butterworth band-pass filters with cut-off frequencies set at 10 and 400 Hz to remove external interference noise and movement artifacts. Integrated EMG (iEMG) obtained each 200 m during the TT was normalized by dividing by the iEMG calculated at the point coinciding with peak torque of the highest MVC. Data processing was performed using MATLAB software.

### Statistical Analysis

The data are presented as means ± SD. Data distribution was analyzed using the Shapiro-Wilk test. two-way ANOVA with repeated-measures were used to verify the effect of condition and distance on the PO, Pan and Paer response. When a significant effect was found, the main effect was analyzed using the least significant difference test for pairwise comparisons. The Effect size (ES) and 95% confidence intervals (95% CI) were calculated to evaluate differences between conditions for mean values of Wtot, Waer, Wan, PO, Pan, Paer and time to cover the TT. The ES was calculated by dividing the difference between mean values of the conditions by the pooled SD [17]. ES of 0.2, 0.6 and 1.2 were considered as small, moderate and large effects, respectively [38]. To make inferences about true (population) values of the effect of caffeine on performance, the uncertainty in the effect was expressed as likelihoods that the true value of the effect represents substantial change (harm or benefit) [38]. The smallest standardized change was assumed to be 0.20. For all analyses, significance was accepted at P<0.05. All analyses were performed using SPSS software (version 13.0; Chicago, IL).

## Results

### Performance Parameters

**Figure 2:**
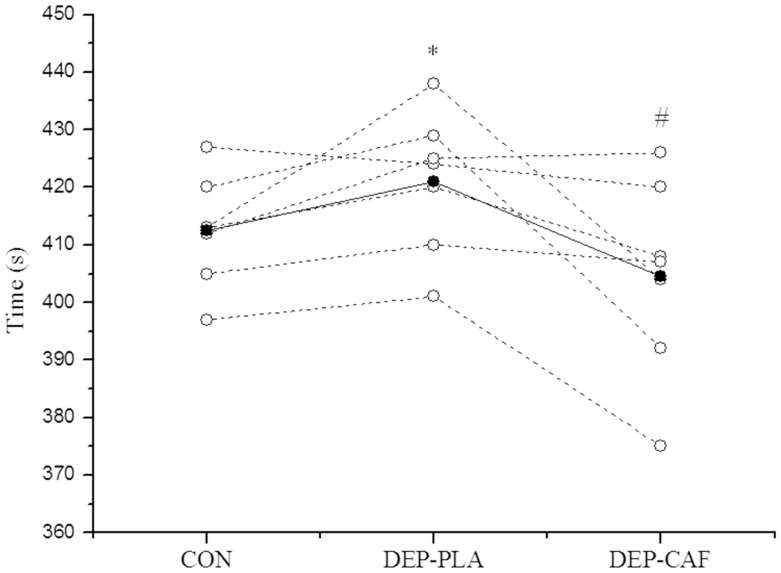
Time to complete the 4-km cycling TT for control (CON), low carbohydrate availability with placebo ingestion (DEP-PLA) and low carbohydrate availability with caffeine ingestion (DEP-CAF). *Moderate effect of DEP-PLA compared to CON and DEP-CAF (ES = 0.65 and 0.94, respectively). #Small effect of DEP-CAF compared to CON (ES = 0.45). Data are expressed as mean (•) and individual (○) values.

Mean and individual values for performance are shown in Figure 2. Only one participant did not improve his performance with caffeine intake (non-responder) compared to the DEP-PLA condition. The mean time to complete the 4-km cycling TT was moderately slower (Fig. 2) in the DEP-PLA (2.1%) than in the CON (ES = 0.65, 95% CI = 0.09–1.22). However, compared with DEP-PLA, caffeine ingestion (DEP-CAF) moderately reduced 4-km completion time by 4.1% (ES = 0.94, 95% CI = 0.10–1.78). The corresponding qualitative inference was 96.7% ‘benefit very likely’. The effect of caffeine (DEP-CAF) compared to CON was small (ES = 0.45, 95% CI = −0.19–1.09), although performance in five of the seven participants was improved in DEP-CAF compared to the CON condition. The corresponding qualitative inference was 79% ‘benefit likely’.

**Figure 3:**
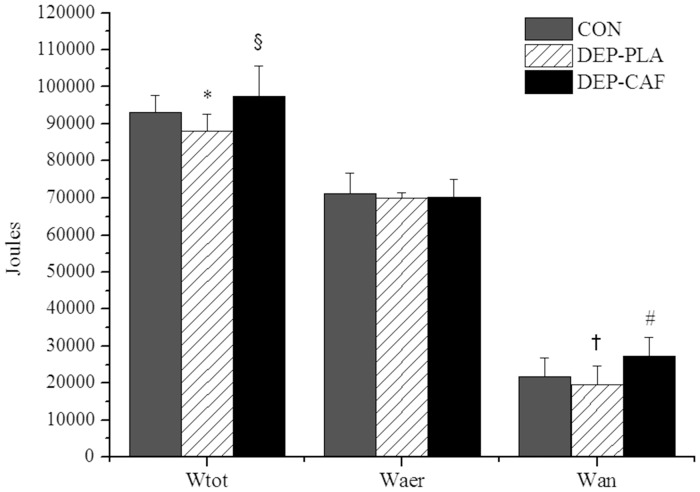
Mean and SD for work total (Wtot), total aerobic (Waer) and anaerobic work (Wan) during the 4-km cycling TT for control (CON), low carbohydrate availability with placebo ingestion (DEP-PLA) and low carbohydrate availability with caffeine ingestion (DEP-CAF). *Moderate effect of DEP-PLA compared to CON (ES = 0.93). §Small and moderate effect of DEP-CAF compared to CON and DEP-PLA (ES = 0.53 and 1.18, respectively). †Small effect of DEP-PLA compared to CON (ES = 0.40). #Moderate and large effects of DEP-CAF compared to CON and DEP-PLA (ES = 0.96 and 1.33, respectively).

Likewise, the mean PO was 7.0 and 10.8% lower (moderate effect), respectively, in DEP-PLA than in CON and DEP-CAF (ES = 0.77, 95% CI = 0.38–1.16and ES = 0.85, 95% CI = 0.09–1.61, respectively). A small effect was found in mean PO between DEP-CAF and CON (ES = 0.28, 95% CI = −0.19–0.76). The Wtot (Fig. 3) performed in the trial was moderately lower in the DEP-PLA (5.6% and 10.7, respectively) than in the CON (ES = 0.93, 95% CI = 0.06–1.80) and DEP-CAF (ES = 1.18, 95% CI = 0.22–2.14), and slightly higher (small effect) in DEP-CAF than CON (ES = 0.53, 95% CI = −0.08–1.14).

When values were expressed every 1-km interval (Table 1), the PO was significantly reduced (P<0.05) in the first kilometer for DEP-PLA, compared with DEP-CAF and CON. In addition, PO at the second kilometer was lower in the DEP-PLA than in the DEP-CAF (P<0.05).

**Table 1:** Mean and SD for Power output (PO), aerobic mechanical power output (Paer), anaerobic mechanical power output (Pan), oxygen uptake (VO_2_)_,_ heart rate (HR), rating of perceived exertion (RPE) and integrated electromyography (iEMG) for each 1-km.

		Distance		
	1-km	2-km	3-km	4-km
**PO (W)**				
CON	245.5±34.2*	217.9±22.3	215.3±27.8	245.6±51.3
DEP-PLA	209.0±19.0	207.5±15.4	202.9±16.7	240.3±42.4
DEP-CAF	255.3±50.5*	237.3±31.3*	222.8±25.5	248.8±34.9
**Paer (W)**				
CON	143.8±11.3	182.2±16.7	186.8±19.8	190.1±18.8*
DEP-PLA	140.9±5.0	173.4±5.6	176.7±6.5	179.5±10.2
DEP-CAF	144.0±15.3	180.5±19.8	184.9±21.8	185.4±22.0
**Pan (W)**				
CON	101.7±40.4	35.7±17.8	28.5±13.2	55.4±44.3
DEP-PLA	68.0±15.8	34.1±10.6	26.2±11.6	60.9±38.0
DEP-CAF	111.4±39.0*	56.8±21.1*	37.9±7.8*	63.4±25.1
V**O_2_ (L.min** ^−**1**^ **)**				
CON	3.2±0.3	4.1±0.3	4.2±0.3	4.2±0.3
DEP-PLA	3.4±0.2	4.1±0.2	4.2±0.2	4.3±0.2
DEP-CAF	3.3±0.3	4.1±0.4	4.2±0.5	4.2±0.5
**HR (bpm)**				
CON	154±20	171±2	175±2	180±2
DEP-PLA	149±19	168±2	172±2	178±3
DEP-CAF	156±19*	174±2*	178±1*	182±2
**RPE (units)**				
CON	11.0±1.7	12.6±0.5	14.6±1.6	16.4±2.4
DEP-PLA	10.7±1.7	12.7±1.3	14.6±1.1	17±1.7
DEP-CAF	10.3±1.5	12.4±1.3	14.9±1.5	17. ±2
**iEMG (%)**				
CON	47.2±6.8	52.0±20.1	46.1±9.2	52.5±12.6
DEP-PLA	45.5±9.0	44.7±6.6	45.2±9.3	51.7±15.8
DEP-CAF	39.3±1.9	41.3±1.7	42.9±2.3	44.7±5.7

iEMG expressed as percentage of EMG value obtained during MVC. CON: control condition; DEP-PLA: low carbohydrate availability with placebo ingestion; DEP-CAF: low carbohydrate availability with caffeine ingestion.

* Significantly higher than DEP-PLA (P<0.05).

### Anaerobic and Aerobic Contribution

Mean Pan during DEP-CAF was moderately greater than in CON (ES = 0.72, 95% CI = 0.089–1.35) and DEP-PLA (ES = 1.18, 95% CI = 0.56–1.78), and slightly lower (small effect) in DEP-PLA than in CON (ES = 0.48, 95% CI = −0.022–0.990). The Pan in DEP-CAF for the first 3 km was significantly higher (P<0.05) than for DEP-PLA, but it was not significantly different from CON (Table 1). No effect was found in the mean Paer during the 4-km cycling TT. However, mean Paer was slightly lower in the last kilometer for DEP-PLA compared with CON (Table 1).

**Figure 4:**
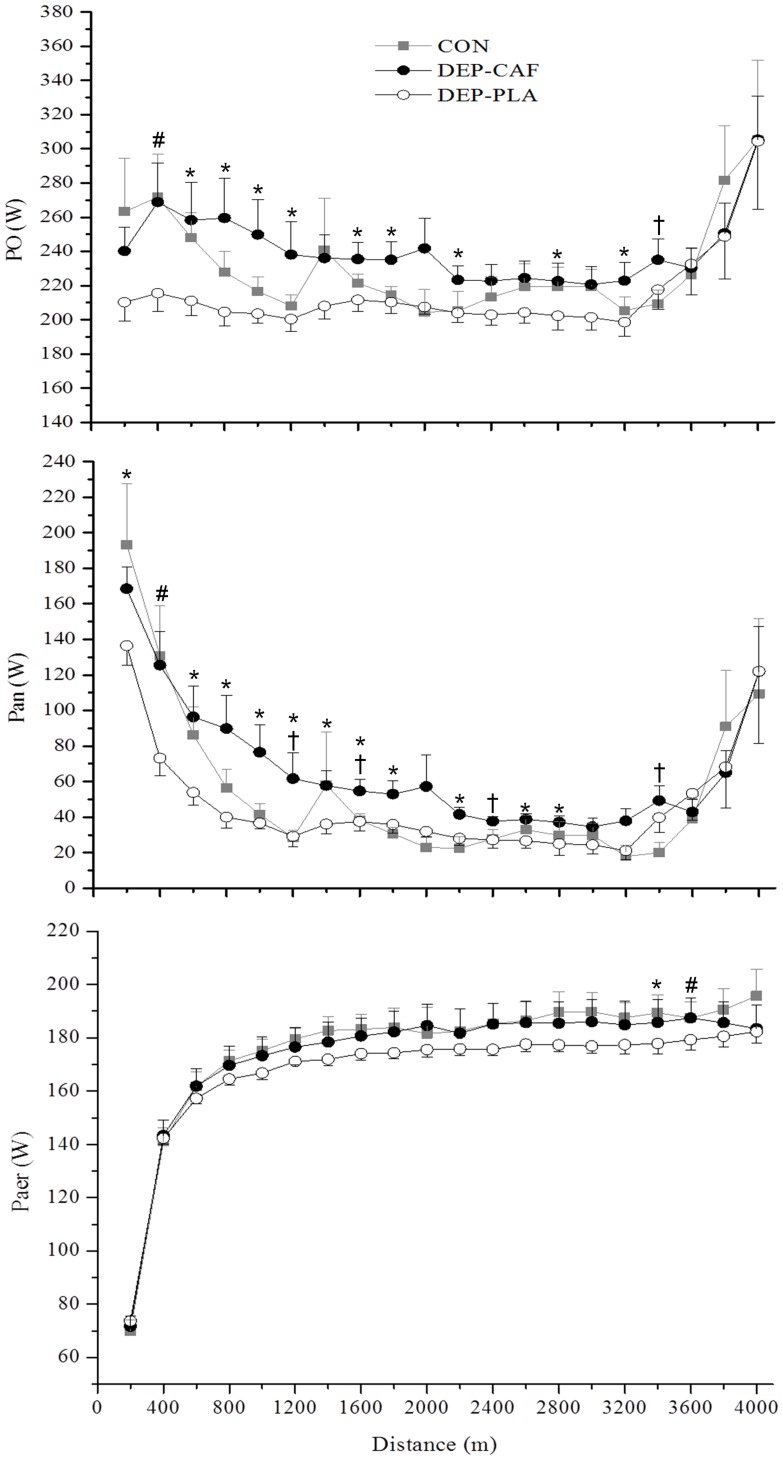
Mean and SD for power output (upper), anaerobic (middle) and aerobic (lower) mechanic power for each 200 m during the 4-km cycling TT for control (CON), low carbohydrate availability with placebo ingestion (DEP-PLA) and low carbohydrate availability with caffeine ingestion (DEP-CAF). *Significantly different between DEP-CAF and DEP-PLA (P<0.05); #Significantly different between CON and DEP-PLA (P<0.05); †Significantly different between DEP-CAF and CON (P<0.05).

Total Waer (Fig. 3) generated during trials was similar between the conditions (DEP-CAF vs CON: ES = 0.17, 95% CI = −0.74–1.08; DEP-CAF vs DEP-PLA: ES = 0.03, 95% CI = −0.49–0.54 and CON vs DEP-PLA: ES = 0.19, 95% CI = −0.40–0.77), whereas there was a moderate to large difference for total Wan between DEP-CAF and CON (ES = 0.96, 95% CI = 0.19–1.73) and DEP-PLA (ES = 1.33, 95% CI = 0.78–1.88), respectively, but the effect was small between CON and DEP-PLA (ES = 0.40, 95% CI = −0.12–0.93). The PO, Pan and Paer profiles are shown each 200-m interval in Figure 4. In general, caffeine ingestion increased mainly PO and Pan compared to DEP-PLA (Fig. 4).

### Perceptual, Physiological and Neuromuscular Responses

There was no difference in the RPE, iEMG or VO_2_ during the 4-km cycling TT between the conditions (Table 1 and 2). However, the HR was significantly higher (P<0.05) in the DEP-CAF than in the DEP-PLA in the first 3 km of the trial (Table 2). No difference was found in HR between CON and DEP-CAF (P>0.05). There was no significant (P>0.05) difference in the resting and pos-TT [La] between conditions (Table 3). Nevertheless, the [La] Pos-TT were significantly (P<0.05) higher than in the rest and Pre-TT in all conditions (Table 3).

**Table 2:** Mean and SD for rating of perceived exertion (RPE), integrated electromyography (iEMG), average oxygen uptake (VO_2_) and heart rate (HR) during the 4-km cycling TT.

Variables	CON	DEP-PLA	DEP-CAF
RPE (units)	13.6±0.7	13.8±0.4	13.8±1.1
iEMG (%)	49.1±9.4	46.8±7.3	42.1±6.9
VO_2_ (L.min^−1^)	3.9±0.3	4.0±0.2	4.0±0.4
HR (bpm)	171±13	168±14	173±14

iEMG expressed as percentage of EMG value obtained during MVC. CON: control; DEP-PLA: low carbohydrate availability with placebo ingestion; DEP-CAF: low carbohydrate availability with caffeine ingestion.

**Table 3:** Mean and SD for lactate concentration at rest, and pre and post the 4-km cycling TT.

	Rest	Pre-TT	Post-TT
**CON**	1.0±0.6	1.0±0.6	9.1±2.9*
**DEP-PLA**	1.1±0.6	0.7±0.2	7.9±1.2*
**DEP-CAF**	1.0±0.3	0.9±0.2	8.8±1.8*

Data are expressed in mmol.L^−1^.

* Significantly higher than Rest and Pre-TT (P<0.05).

## Discussion

The main finding of the present study was that acute caffeine ingestion reversed the impairment in time-trial performance following a protocol designed to lower endogenous CHO availability. Furthermore, the reestablishment of the performance with caffeine ingestion was associated with an increase in the Pan and total anaerobic work generated during the 4-km cycling TT, indicating that caffeine allows access to an anaerobic “reserve” that is not normally used. The increase in the performance with caffeine ingestion seems to be explained by a higher PO and anaerobic contribution during the first 2–3 km of the trial.

### Effect of Caffeine on 4-km Cycling TT Performance

Previous studies have reported an increase in high-intensity exercise performance after caffeine ingestion [10]–[12]. Recently, Wiles et al. [13], using the same caffeine dose as the present study (5 mg.kg^−1^), reported an improvement (3.1%) in 1-km cycling TT performance compared to a placebo. This improvement was accompanied by a 3.6% increase in the mean PO [13]. In the present study, we observed a 7.0% reduction in mean PO and a 2.1% increase in the time to complete the trial when TT was performed after an exercise protocol designed to reduce CHO availability, in comparison with CON. However, caffeine intake increased mean PO by 10.8% and reduced the time to cover the 4-km TT by 4.1%, when compared with DEP-PLA. When compared with control, caffeine intake increased mean PO by 4.3% and reduced the time to complete the trial by 1.9%. Qualitative inferences indicated that a benefit was ‘very likely’ and ‘likely’ when DEP-CAF was compared to DEP-PLA and CON, respectively, suggesting that performance improvements of 4.1 and 1.9% are meaningful for our participants. Our study demonstrates for the first time that, even with a reduced CHO availability, caffeine intake restores time-trial performance to levels found when endogenous CHO availability is normal.

Athletes adopted a more conservative starting pacing strategy when CHO stores were depleted. It is not fully clear if this conservative starting was caused by an intramuscular effect of the muscle glycogen depletion and/or a psychological strategy. However, despite the subjects being aware that they were depleted, they were not able to identify which capsule (caffeine or placebo) had subsequently been ingested, and, even then, adopted a more aggressive pacing strategy in DEP-CAF than DEP-PLA. Both iEMG and RPE at the beginning of the DEP-PLA trial were also similar to the DEP-CAF trial, even with a lower PO, suggesting that any effect of manipulation may have happened in the muscle. Furthermore, caffeine supplementation restored the PO in the first two kilometers and it was not associated with a reduced PO in the rest of the time trial.

In contrast to the results of the present study, Hettinga et al. [24] reported that a higher PO in the first 2 km of a 4-km cycling TT results in an impairment of the PO in the second half of the trial. It should be noted however, that the pacing strategy during the first 2 km in the Hettinga study was dictated by the researchers, and the participants were “enforced” to perform a constant PO at 105% above the mean PO until the end of the second kilometer. This “enforced”, constant-paced exercise during the first 2 km may have induced a greater physiological strain than self-paced exercise, and provoked a reduction in PO during the second half of the trial [39], [40]. Therefore, caffeine seems to attenuate the decrement in power output observed early in CON and DEP-PLA conditions, and preserves the ability to optimally perform the second half of the trial.

Even though mean PO was higher in the first 2 km of DEP-CAF compared with DEP-PLA, the RPE was not significantly different between the conditions, suggesting that participants were able to perform the first 2 km of a 4-km cycling TT with a higher PO/RPE ratio when caffeine was ingested. This is in accordance with previous results suggesting that, independent of the physiological or metabolic status, athletes normally adopt a pattern of increase in RPE proportional to the exercise distance completed [41]–[43]. For example, positive (e.g. nutritional supplementation) and negative (e.g. hypoxia) changes in the homeostatic status throughout the TT have been reported to provoke an increase or reduction in PO, respectively, in order to maintain the same RPE template during exercise trials [42]–[44]. Caffeine may influence RPE via a direct blockade of adenosine A2a receptors in the brain [45], permitting more external work to be performed for a given conscious perception. However, although any alteration in RPE with caffeine should have increased motor drive and neuromuscular excitability [18], the iEMG was not altered in the present study. Alternatively, caffeine may have had a peripheral effect on increasing muscle function, which, in turn, would reduce muscle sensory signals to the brain and consequently reduce RPE.

### Effect of Caffeine on Anaerobic and Aerobic Contribution

According to some studies [2], [5], endogenous CHO depletion reduces the contribution of the anaerobic system, possibly due to a limitation in the rates of glycogenolysis and glycolysis. In fact, Blomstrand and Saltin [46], using a protocol to reduce endogenous CHO availability in one leg, showed that the breakdown of muscle glycogen during 60 minutes of exercise at 75% VO_2_max was ∼ 48% less in the leg that started the exercise with low muscle glycogen (99±18 µmol.kg dry wt^−1^) than the leg that began with normal muscle glycogen levels (207±22 µmol.kg dry wt^−1^). When analyzing each km, we observed an increase in the Pan during the first 3 km for DEP-CAF compared to the DEP-PLA. This large anaerobic production early in the trial was accompanied by a significant increase in the PO, supporting the idea that power distribution during a TT appears to be regulated primarily by changes in the anaerobic contribution [23], [24]. In related results, Hettinga el al. [24] reported that the Pan was higher during the first 2 km of a 4-km cycling time trial when athletes were asked to perform the first 2 km at a supra-mean PO intensity (105% of the mean PO), but was significantly reduced in the second half of the trial when the athletes were free to reduce the PO. These results are consistent with those reported recently by Aisbett et al. [23] who induced fast-, even-, and slow-starting during the first 25% of a 5-min cycling time trial (approximating the duration of a 4000-m cycling TT), and observed that the oxygen deficit (anaerobic contribution) was greater in the first quarter and lower in the second and third quarters of exercise in the fast-start trial, compared with the two other pacing strategies. In contrast, we found that despite a higher Pan in first 3 km after caffeine ingestion, the Pan in the last km was not impaired when compared with the other conditions, suggesting that caffeine maintained the Pan throughout the trial and promoted an increase in the total anaerobic contribution.

Several studies have suggested that the total amount of anaerobic energy that can be produced during a TT is fixed [23]–[25]. Indeed, Hettinga et al. [25] demonstrated that despite inducing their participants to perform the first 750 m of a 1500-m cycling TT at 105% (supra) and 95% (sub) of the mean PO measured during the even-paced time trial, the total amount of anaerobic work generated over the time trial remained unchanged. Similarly, Aisbett et al. [23] also reported no difference in the total energy provided by the anaerobic system when the cyclists performed a 5-min cycling TT using different starting strategies. These results suggested that the pattern of anaerobic energy distribution can vary with pacing strategy, but that the total anaerobic capacity remains fixed. On the other hand, it has been reported that caffeine intake promotes an increase in the quantity of anaerobic energy produced during high-intensity, time-to-exhaustion exercise [10]–[12], although this has not been measured during closed-loop exercise such as a TT.

In the present study, we found that the total amount of anaerobic energy was higher in DEP-CAF than in both DEP-PLA and CON, indicating that caffeine exerts a more potent effect on the anaerobic contribution than low CHO availability. Furthermore, the greater Wan during the 4-km cycling TT after caffeine ingestion indicates that the total amount of anaerobic energy expenditure during TT exercise may not be fixed and that caffeine allows access to an anaerobic “reserve” that is not used under normal conditions. The existence of an anaerobic reserve has been demonstrated recently by Corbett et al. [47], who reported that the total anaerobic energy yield during a 2000-m cycling TT was higher when the participants believed that they were competing against another athlete of similar ability (head-to-head), than when they exercised alone (time trial), suggesting that a motivational stimulus promotes the use of a greater degree of the anaerobic “reserve”. To the best of our knowledge, the present study is the first to demonstrate that caffeine intake increases the total anaerobic work produced during a middle-distance cycling TT, even though the athletes started the trial with a low CHO availability. In addition, although PO and Pan were increased at the beginning and in the middle of the TT with caffeine ingestion, when compared to the DEP-PLA condition, there were no differences in the last 600 m between the conditions, and this was accompanied by a similar [La] Post-TT, suggesting that CAF was not able to increase the anaerobic contribution at the end of the trial.

The mechanism by which caffeine ingestion exerts its effect on the anaerobic contribution remains unclear. Simmonds et al. [12] suggested that caffeine increases the anaerobic contribution through its antagonistic action on the adenosine receptors, which could unlock any inhibitory effect of adenosine on phosphofructokinase enzyme activity in skeletal muscle. Furthermore, Bridge and colleagues [16] have suggested that caffeine ingestion results in an increase in the release of calcium, facilitating the conversion of the enzyme phosphorylase *b* to it more active form *a*, which would lead to an acceleration of glycogenolysis. However, this last mechanism seems unlikely *in vivo*, since a greater mobilization of calcium from sarcoplasmic reticulum has been observed only at high concentrations of caffeine that would be toxic to humans [48].

### Effects of Caffeine on Neuromuscular Responses

It has been hypothesized that caffeine intake improves performance during high-intensity exercise through an increase in electromyography activity [18]. An increase in both EMG activity and performance during short-duration, maximal-dynamic contractions has been demonstrated after caffeine ingestion [18]. In the present study however, although the PO was greater with caffeine intake, there was no difference in iEMG between conditions. These conflicting results could possibly be explained by the different protocols adopted and the muscles analyzed. While we used a cycling time-trial test, and measured the EMG of the vastus lateralis muscle, Bazzucchi et al. [18] assessed elbow flexion torque with an isokinetic dynamometer and EMG was recorded from the biceps brachii. On the other hand, our results are consistent with findings from other studies [49], [50] that observed no significant differences in the EMG recorded from the vastus lateralis muscle during isometric contractions after caffeine ingestion. However, the measurement and interpretation of EMG during dynamic exercise is difficult and may not be sufficiently sensitive to measure small changes in muscle activation [49]. Therefore, conclusions about the effects of caffeine on iEMG during dynamic exercise have to be interpreted with caution. Nevertheless, in the absence of other methods to quantify muscle activation levels during dynamic exercise, changes in iEMG amplitude are the only way to indirectly measure muscle activation levels during a cycling time trial. Thus, while we interpret our iEMG data with caution, our findings indicate that iEMG activity did not change with caffeine intake.

A potential limitation is that we did not include a control session (without CHO depletion) with caffeine ingestion. Despite the fact that this could have provided a more complete knowledge about the effects of caffeine on pacing strategy and TT performance with different levels of endogenous CHO availability, the inclusion of more experimental sessions had the potential to reduce the motivation of participants to participate in so many trials, potentially affecting our performance results. Thus, we focused the present study on verifying if caffeine could reverse the deleterious effect of low CHO availability on performance. In addition, the standardisation of the starting gear and the prevision of verbal instead visual distance feedback should also be acknowledged. However, the hard gear ratio as the used in the present study (53×16) has been described in the literature and justified due the high-intensity, fast-start nature of the 4-km cycling TT [20]. Verbal instead of visual distance feedback has also been used in previous research [20]. Finally, we were unable to blind the participants to the reduced CHO availability and this may have influenced pacing in the trials. While blinding the participants to the CHO availability in the diet is practically impossible, we cannot fully discard the possibility of any conscious alteration in pacing due to awareness of being in a low-CHO state. However, this could be expected to affect pacing equally in both CHO-depletion conditions, but the participants paced themselves faster in DEP-CAF than DEP-PLA, while iEMG signal was not altered, suggesting that pacing modifications during depleted-CHO condition may have not been “consciously” determined.

### Conclusions

In conclusion, the results from the present study have shown that acute ingestion of caffeine induced a higher mean PO and anaerobic contribution during the first 2–3 km of the 4-km time trial. Furthermore, caffeine seems to attenuate the decrement in power output observed early in CON and DEP-PLA conditions, and preserves the ability to optimally perform the second half of the trial. In addition, caffeine ingestion reversed the impairment in time-trial performance caused by low CHO availability to levels found when endogenous CHO availability is normal. This improvement in performance was associated with a greater total anaerobic energy contribution with caffeine ingestion compared to both DEP-PLA and CON, indicating that caffeine exerts a more potent effect on the anaerobic contribution than low CHO availability. Therefore, we suggest that the total amount of anaerobic energy expenditure during TT exercise may not be fixed and that caffeine ingestion (even following a protocol to reduce endogenous CHO availability) allows access an anaerobic “reserve” that is not accessed under normal conditions. Our results suggest that caffeine may have an effect on intramuscular metabolism, i.e. increase on anaerobic contribution and total anaerobic work, rather than any significant effect on muscle recruitment.
